# Protection against prolonged pneumococcal infection involves structural changes in C-reactive protein and subsequent binding to both phosphocholine and amyloids on the bacterial surface

**DOI:** 10.3389/fimmu.2025.1631409

**Published:** 2025-07-16

**Authors:** Alok Agrawal, Donald N. Ngwa, J. Paul Simons, Sanjay K. Singh

**Affiliations:** ^1^ Department of Biomedical Sciences, College of Medicine, East Tennessee State University, Johnson City, TN, United States; ^2^ Centre for Amyloidosis and Acute Phase Proteins, Division of Medicine, University College London, London, United Kingdom

**Keywords:** C-reactive protein, *Streptococcus pneumoniae*, bacterial amyloids, complement evasion, serum amyloid P component

## Abstract

C-reactive protein (CRP) protects mice during the initial stages of *Streptococcus pneumoniae* infection. In order to be protective against all stages of infection, we hypothesize that CRP binds to two different ligands on pneumococci. In its native form, CRP binds to phosphocholine residues of C-polysaccharide to activate complement. In its altered form, CRP binds to amyloid-like structures (amyloids) formed on complement inhibitors recruited by pneumococci. We employed CRP knockout mice to test this hypothesis. In one approach, both wild-type CRP and E42Q/F66A/T76Y/E81A mutant CRP (E-CRP-1) were administered together. E-CRP-1 does not bind to phosphocholine but binds to amyloids. In another approach, Y40F/E42Q mutant CRP (E-CRP-2) was administered. E-CRP-2 binds to both phosphocholine and amyloids. When CRP was administered to mice 12 h after inoculation, then unlike wild-type CRP by itself, the combination of wild-type CRP and E-CRP-1 was protective and E-CRP-2 alone was protective. We also detected amyloids on pneumococci. The serum levels of the amyloid-binding protein, serum amyloid P component (SAP), were higher in CRP knockout mice than in wild-type mice. Also, the basal SAP levels were higher in female than in male mice and, conversely, male mice were more susceptible than female mice to severe infection. We conclude that the protection against prolonged pneumococcal infection requires structural changes in CRP and binding to both phosphocholine and amyloids on pneumococci. The sources of amyloids can be virulence factors or recruited complement inhibitors or both. Combined data also raise the possibility that SAP cooperates with CRP in reducing bacteremia and bacterial load.

## Introduction

C-reactive protein (CRP) is an evolutionarily conserved protein, which suggests that CRP performs host-defense functions in all organisms from arthropods to humans (1–4). In humans, CRP is a component of the acute phase response; the serum level of CRP increases thousand-fold or more in acute inflammatory states (5). CRP is composed of five identical subunits arranged in a cyclic pentameric symmetry (6). CRP binds to phosphocholine (PCh)-containing substances, such as C-polysaccharide of the cell wall of *Streptococcus pneumoniae*, in a Ca^2+^-dependent manner (7). All five subunits of CRP have a PCh-binding site consisting of amino acid residues Phe^66^, Thr^76^ and Glu^81^ (8–10). PCh-complexed CRP activates the classical pathway of the complement system, leading to the destruction of the ligand (11). Human CRP activates mouse complement system also and therefore mice are widely used to investigate the *in vivo* functions of CRP (12–14).

The native pentameric conformation of CRP is altered under experimental inflammatory conditions such as in the presence of acidic pH or reactive oxygen species (15–18). At acidic pH, CRP has been shown to bind to amyloid-β peptide 1-42 (Aβ) (16, 19, 20). Some proteins, when immobilized, express Aβ-like structures (amyloids), and acidic pH-treated CRP binds to such immobilized proteins through the exposed amyloids (21, 22). The existence of non-native CRP *in vivo* and their deposition at sites of inflammation have been demonstrated by employing antibodies specific for non-native CRP (23, 24); however, it is not evident whether CRP seen at the sites of inflammation was monomeric CRP or non-native pentameric CRP. Like the PCh-binding function of CRP, the amyloid-binding function of CRP has also been conserved throughout evolution (25).

**Figure 1:**
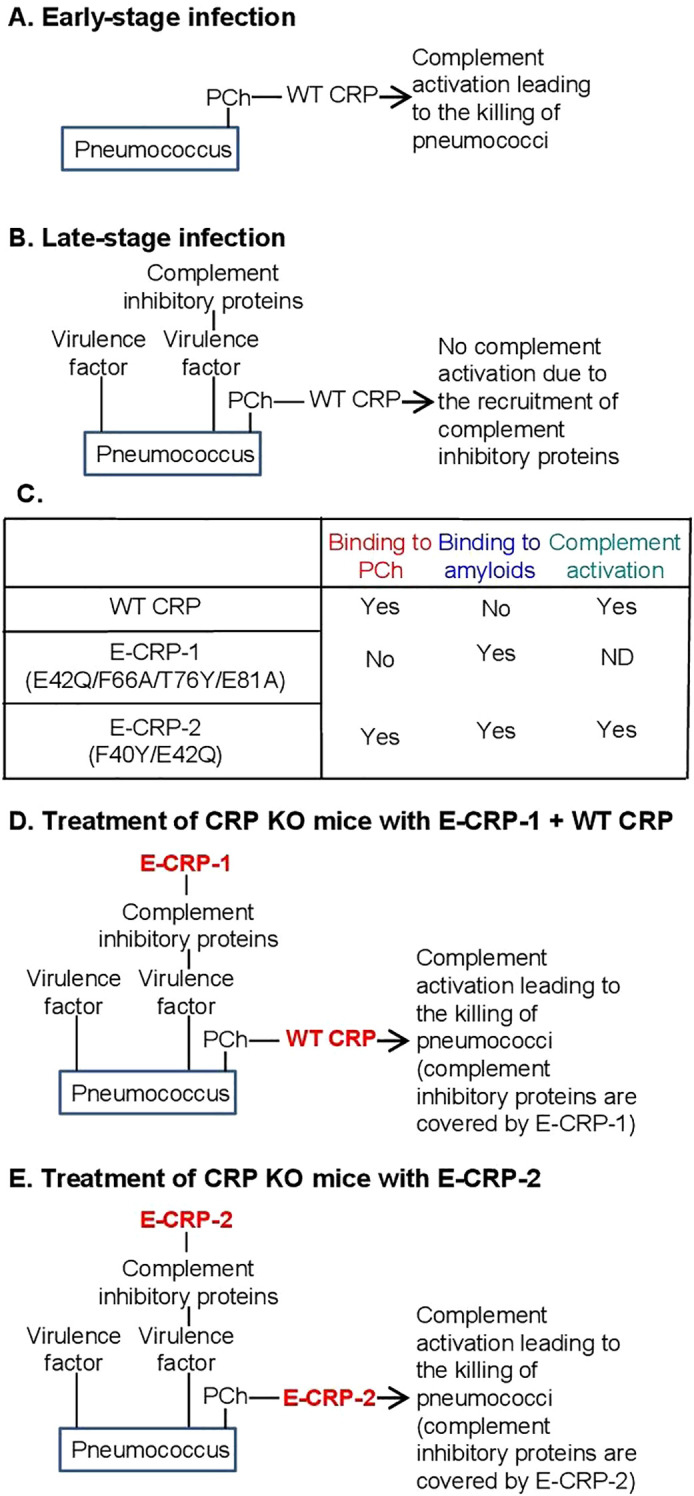
**(A, B)** Suggested mechanism of action of CRP in pneumococcal infection. **(C)** Ligand-recognition and effector functions of CRP molecules employed in this study. **(D, E)** Hypothesis for the mechanism of action of exogenously administered CRP in protecting CRP KO mice against prolonged pneumococcal infection.

Human CRP protects against lethal pneumococcal infection by decreasing bacteremia in mouse models of the disease (26–30). Complement activation by ligand-complexed CRP is necessary for CRP-mediated protection against infection (31–33). In mouse models in which human CRP was passively administered to mice, CRP was protective only when administered 6 h before to 2 h after inoculation with pneumococci (34). It has been shown that pneumococci recruit complement-inhibitory proteins on their surface to become complement attack-resistant (35–42). The presence of complement-inhibitory proteins on pneumococci could be the reason for the inability of CRP to protect when administered 2 h after inoculation (**Figures 1A, B**).

It remains to be established that the ability of structurally altered pentameric CRP to bind to amyloids contributes to protection against pneumococcal infection (43). Since the acidic pH-induced changes in CRP are reversible at physiological pH, acidic pH-treated CRP cannot be administered to mice for *in vivo* studies (16). Therefore, recombinant CRP mutants have been created that mimic the amyloid-binding property of acidic pH-treated CRP (20, 21, 44). Previously, two such CRP mutants have been used as tools to investigate the host-defense functions of structurally altered CRP *in vivo*: E-CRP-1 and E-CRP-2 (22, 43). The ligand-recognition and effector functions of wild-type (WT) CRP, E-CRP-1 and E-CRP-2 are summarized in **Figure 1C**. E-CRP-1 (E42Q/F66A/T76Y/E81A CRP mutant) does not bind to PCh due to the mutations in the PCh-binding site (43, 45). E-CRP-1, however, binds to amyloids due to the presence of the E42Q mutation (20, 21). In contrast to E-CRP-1, E-CRP-2 (Y40F/E42Q CRP mutant) retains the ability to bind to PCh and, in addition, also binds to amyloids (20, 21, 43, 46). Biochemical analyses of acidic pH-treated WT CRP and of CRP mutants suggested that CRP gains the amyloid-binding property due to the loss of one Ca^2+^ from CRP and that the cholesterol-binding region of CRP which contains the Ca^2+^-binding site may be involved (16, 20, 21, 47).

Our earlier findings that immobilized proteins express amyloids (21) prompted us to hypothesize that complement inhibitory proteins recruited by pneumococci may express amyloids. It is not known whether the virulence factors present on pneumococci also express amyloids. In this study, we tested the overall hypothesis that if the amyloid-expressing proteins on the pneumococcal surface are blocked by E-CRP-1/-2, then WT CRP should be able to protect mice against all stages of pneumococcal infection; for example, WT CRP should be able to protect mice against infection even when administered to mice 12 h post-inoculation. Two approaches were employed in this study. In the first approach, both WT CRP (for PCh-binding) and E-CRP-1 (for amyloid-binding) were administered to mice. In this approach, E-CRP-1 can bind to amyloid-expressing proteins and PCh-bound WT CRP can then activate complement and kill the bacteria (**Figure 1D**). In the second approach, E-CRP-2 (for both PCh-binding and amyloid-binding) was administered to mice, assuming that some E-CRP-2 molecules would bind to amyloids and some to PCh (**Figure 1E**). PCh-bound E-CRP-2 can then activate complement and kill the bacteria. CRP knockout (KO) mice were used in this study since our hypothesis could only be tested in CRP-deficient mice.

## Materials and methods

### Preparation of CRP

The cDNAs for E42Q/F66A/T76Y/E81A mutant CRP (E-CRP-1) and Y40F/E42Q mutant CRP (E-CRP-2) were constructed, expressed in CHO cells using the ExpiCHO Expression System (Thermo Fisher Scientific) and purified from the cell culture supernatants, as described previously (43, 46, 48). E-CRP-1 was purified by employing Ca^2+^-dependent affinity chromatography on a phosphoethanolamine-conjugated Sepharose column, followed by ion-exchange chromatography on a MonoQ column and gel filtration on a Superose12 column. E-CRP-2 from cell culture supernatants and native WT CRP from discarded human pleural fluid were purified by employing Ca^2+^-dependent affinity chromatography on a PCh-conjugated Sepharose column, followed by ion-exchange chromatography on a MonoQ column and gel filtration on a Superose12 column. The purity of CRP preparations was confirmed by denaturing 4-20% SDS-PAGE under reducing conditions. Purified CRP was dialyzed against 10 mM Tris-HCl, pH 7.2, containing 150 mM NaCl and 2 mM CaCl_2_, and was subsequently treated with Detoxi-Gel Endotoxin Removing Gel (Thermo Fisher Scientific). The concentration of endotoxin in CRP preparations was determined by using the Limulus Amebocyte Lysate kit QCL-1000 (Lonza). Purified CRP was stored at 4°C and used within a week.

### Pneumococci

*S. pneumoniae* type 3, strain WU2, was obtained from Dr. David Briles (University of Alabama at Birmingham, Birmingham, AL, USA) and used as described previously (43, 45). In brief, pneumococci were made virulent by sequential i.v. passages in mice and were stored in aliquots at -80°C. For each experiment, a separate aliquot of pneumococci was thawed and cultured. Cultured pneumococci were resuspended in normal saline and the concentration of pneumococci (cfu/ml) was adjusted based on the absorbance of the suspension at 600 nm (A_600_ = 1.00 = 1.2 x 10^9^ cfu/ml). Within 2 h, 100 µl of pneumococci suspension containing the required number (cfu) of pneumococci, as mentioned in the figures, was injected into mice. The concentration of pneumococci was confirmed next day by plating.

### Mice

The method for the generation of pure-line C57BL/6 CRP KO mice has been previously described (49). The breeding colony of CRP KO mice was maintained in the Division of Laboratory Animal Research of our university. Male and female WT C57BL/6 mice were purchased from Jackson Laboratories. Mice were brought up and maintained according to protocols approved by the University Committee on Animal Care. Mice were 8–10 weeks old when used in experiments.

### Mouse protection experiments

Mouse protection experiments were performed exactly as described previously (29, 43). In brief, mice were inoculated with pneumococci; the numbers of pneumococci (cfu) are mentioned in the figures. CRP (25 μg) was administered at different time points as mentioned in the figures. The amount of endotoxin in 25 μg of all CRP preparations was <1.0 endotoxin units. Survival of mice was recorded three times per day for 7 days. Survival curves were generated using the GraphPad Prism 9 software. To determine *p*-values for the differences in the survival curves among various groups, the survival curves were compared using the software’s Logrank (Mantel-Cox) test.

To determine bacteremia in the surviving mice, blood was collected daily for 5 days from the tip of the tail vein, diluted in normal saline, and plated on sheep blood agar for colony counting. The bacteremia value for dead mice was recorded as 10^9^ cfu/ml because mice died when the bacteremia exceeded 10^8^ cfu/ml. The scatter plots of the bacteremia data and the median bacteremia value for each group were generated using the GraphPad Prism 9 software. The software’s Mann-Whitney test was used to determine *p*-values for the differences in bacteremia among various groups at each time point. The median values shown in the scatter plots for each group of mice were also plotted separately for easier comparison of all groups of mice in a single figure.

### Assay for the detection of amyloids on pneumococci

Microtiter wells (Corning, 9018) were coated with increasing numbers of pneumococci in 100 µl TBS, pH 7.2, in duplicate, and incubated overnight at 4°C. The unreacted sites in the wells were blocked with TBS containing 0.5% gelatin for 45 min at room temperature. Both, polyclonal anti-Aβ antibodies (Novus, NBP2-25093) and monoclonal anti-Aβ antibodies (Novus, NBP2-13075) were used to detect the amyloids on the coated pneumococci. Normal rabbit IgG and normal mouse IgG were used as controls for the antibodies. The anti-Aβ antibodies (10 μg/ml) diluted in TBS containing 0.1% gelatin and 0.02% Tween 20 were added to the wells and incubated at 37°C for 1 h. After washing the wells, bound polyclonal anti-Aβ antibodies were detected by using HRP-conjugated donkey anti-rabbit IgG (GE Healthcare) and bound monoclonal anti-Aβ antibodies were detected by using HRP-conjugated goat anti-mouse IgG (Thermo Fisher Scientific). Color was developed, and the OD was read at 405 nm.

### Assays for the measurement of mouse CRP and serum amyloid P component

The levels of mouse CRP (moCRP) in the sera obtained from the blood of WT mice were measured by using Mouse CRP Quantikine ELISA Kit (R&D, catalog number MCRP00). The levels of mouse serum amyloid P component (SAP) in the sera from WT and KO mice were measured by using Mouse SAP Quantikine ELISA Kit (R&D, catalog number MPTX20).

## Results

### Male mice are more susceptible than female mice to infection

To establish a CRP KO mouse model of infection with *S. pneumoniae* type 3, strain WU2, we titrated the dose of bacteria needed for inoculation. In order to determine whether there was any difference between WT and KO mice for their susceptibility to infection and whether there was any difference between male and female mice, both WT and KO mice and both male and female mice were employed. The median survival time (MST, the time taken for the death of 50% of mice) for each dose of bacteria was then determined.

**Figure 2:**
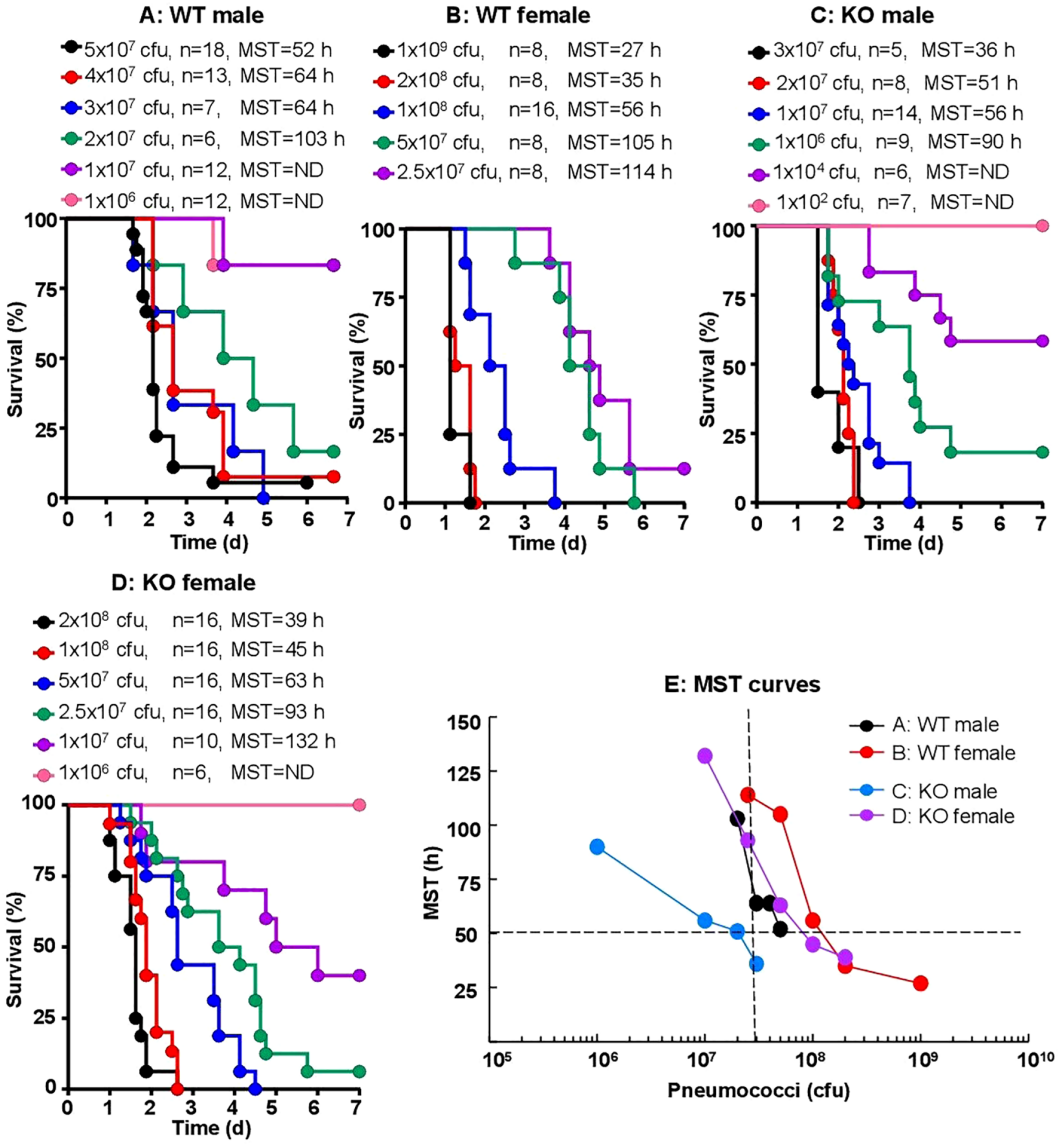
Sex-specificity and susceptibility of mice to infection. Mice were injected with 10^2–^10^9^ cfu of pneumococci, as shown for each group of mice (n, number of mice). The MST values are shown for each dose of pneumococci for each group of mice (ND, MST not determined since >50% mice survived). The data are combined from two separate experiments with six to nine mice for each dose of pneumococci in each group of mice. **(A)** Survival of male WT mice. **(B)** Survival of female WT mice. **(C)** Survival of male CRP KO mice. **(D)** Survival of female CRP KO mice. **(E)** The MST values for each group of mice shown in A-D are plotted together for comparing the susceptibility of all four types of mice. To determine *p*-values for the differences in the MST curves, the curves were subjected to two-way ANOVA followed by Tukey’s multiple comparison test. The *p*-values for the differences in the MST curves between groups A B, C D, A C and B D were 0.07, 0.01, 0.03 and 0.31, respectively.

As shown in the survival curves for all four types of mice (**Figures 2A–D**), and as expected, the MST decreased as the inoculation dose of bacteria increased. To determine the relative susceptibility of each type of mice to infection in terms of the dose of bacteria and the corresponding survival times, the data (**Figures 2A–D**) were compiled together and presented as MST curves (**Figure 2E**). The survival time of 50 h and the dose of 3 x 10^7^ cfu of bacteria were chosen for comparing the four MST curves. As shown, for each type of mice to survive for 50 h after inoculation, the doses of bacteria were 13 x 10^7^, 8 x 10^7^, 5 x 10^7^ and 2 x 10^7^ cfu for WT female, KO female, WT male and KO male mice, respectively. Similarly, for mice inoculated with 3 x 10^7^ cfu of bacteria, the survival times were 112 h, 86 h, 63 h and 36 h for WT female, KO female, WT male and KO male mice, respectively. These results and the statistical analyses of the MST curves indicated that male mice were significantly more susceptible than female mice to infection. Also, CRP KO mice were more susceptible than WT mice to infection, although the difference between the MST curves for female WT and female KO mice was not statistically significant.

CRP KO male mice inoculated with 10^7^ cfu of pneumococci were employed as the mouse model for all CRP-mediated protection experiments, unless otherwise mentioned.

### KO mice are most protected when WT CRP is administered prior to or within an hour of inoculation

**Figure 3:**
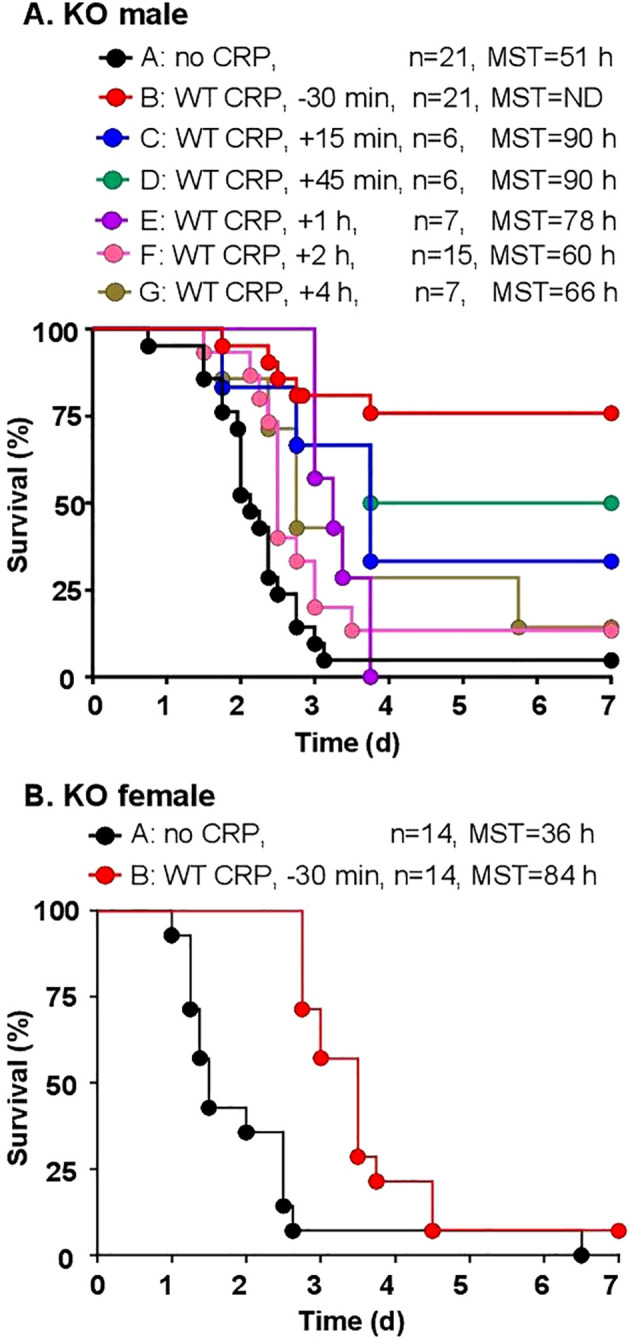
Survival of CRP KO mice infected with pneumococci and treated with WT CRP. The data are combined from two to three separate experiments with six to eight mice in each group in each experiment (n, number of mice). The MST values are shown for each group of mice (ND, MST not determined since >50% mice survived). **(A)** Male CRP KO mice. CRP was injected at various time points in different groups of mice, from 30 min before to 4 h after inoculation with pneumococci (10^7^ cfu). The *p*-value for the difference in the survival curves between groups A and B was <0.001. The *p*-values for the differences between A C, A D, A E and A F were 0.01. The *p*-value for the difference between A and G was 0.05. The *p*-values for the differences between B C and B D were >0.05. The *p*-values for the differences between B E, B F and B G were <0.005. **(B)** Female CRP KO mice. CRP was injected 30 min prior to inoculation with pneumococci (10^8^ cfu). The *p*-value for the difference between groups A and B was <0.05.

The protective effects of WT CRP administered 30 min prior to and 15 min to 4 h after inoculation into KO male mice (**Figure 3A**) were determined first, by analyzing the survival curves of mice. Statistical analysis of the data showed that, when compared to group A mice (bacteria alone), CRP was clearly protective for groups B-F of mice, that is, if injected 30 min before to 2 h after inoculation. The *p*-value between group A and group G (CRP injected 4 h after inoculation), however, was 0.05, suggesting that CRP may still be protective. When compared to group B mice in which CRP was administered 30 min prior to inoculation, CRP was found to be protective only when injected 45 min after inoculation, but not later. Overall, as shown, as the interval between inoculation and CRP administration increased, the MST decreased. Mice survived longest and the MST could not be determined when CRP was given to mice 30 min before inoculation. Mice survived shortest when CRP was given to mice 4 h after inoculation. These results were similar to previously published data on the effects of WT CRP in WT mice: CRP was protective only when administered either prior to inoculation or at most 2 h post-inoculation. To confirm that WT CRP was protective against infection in KO female mice also, the 30 min time point for CRP injection prior to inoculation was chosen. As shown in **Figure 3B**, CRP protected female mice also. In all subsequent protection experiments, only CRP KO male mice were employed.

### WT CRP and E-CRP-1 together protect KO mice even when administered 12 h after inoculation

**Figure 4:**
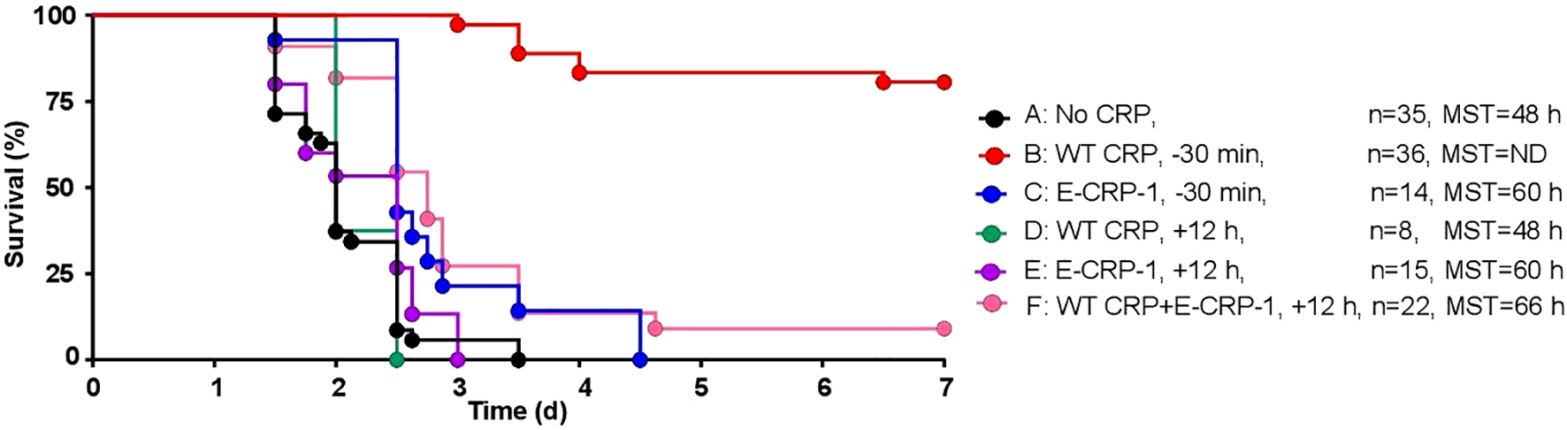
Survival of CRP KO mice infected with pneumococci and treated with E-CRP-1. The data are combined from two to four separate experiments with six to nine mice in each group in each experiment (n, number of mice). The MST values are shown for each group of mice (ND, MST not determined since >50% mice survived). CRP was injected 30 min prior to inoculation with pneumococci (10^7^ cfu) in groups B and C and 12 h after inoculation in groups D-F. The *p*-values for the differences in the survival curves between groups A B, A C and A F were <0.001. The *p*-values for the differences between A D and A E were >0.05. The *p*-values for the differences between B C, B D, B E and B F were <0.001. The *p*-values for the differences between C D, C E and C F were 0.004, 0.04 and 0.46, respectively. The *p*-value for the difference between E and F was 0.01.

We determined the protective effects of E-CRP-1 which does not bind to PCh and instead binds to amyloids. WT CRP administered 30 min prior to inoculation was included as a control for the animal model. As shown in **Figure 4**, and as has been reported earlier (43), WT CRP protected mice when administered 30 min prior to inoculation (group B) but did not protect when administered 12 h after inoculation (group D). The survival curve of mice treated with E-CRP-1, 30 min prior to inoculation (group C), was found to be significantly different from both group A (no CRP) and group B. However, like WT CRP, E-CRP-1 was not protective when administered 12 h after inoculation (group E). Although WT CRP and E-CRP-1 were not protective when either one was administered 12 h after inoculation, the survival curve of mice treated with the combination of WT CRP and E-CRP-1 was significantly different from both groups D and E and also different from group A. The survival times of mice treated with both WT CRP and E-CRP-1 together were longer than the survival times of mice treated with either WT CRP or E-CRP-1 alone and 10% of mice survived at the end of the experiment.

**Figure 5:**
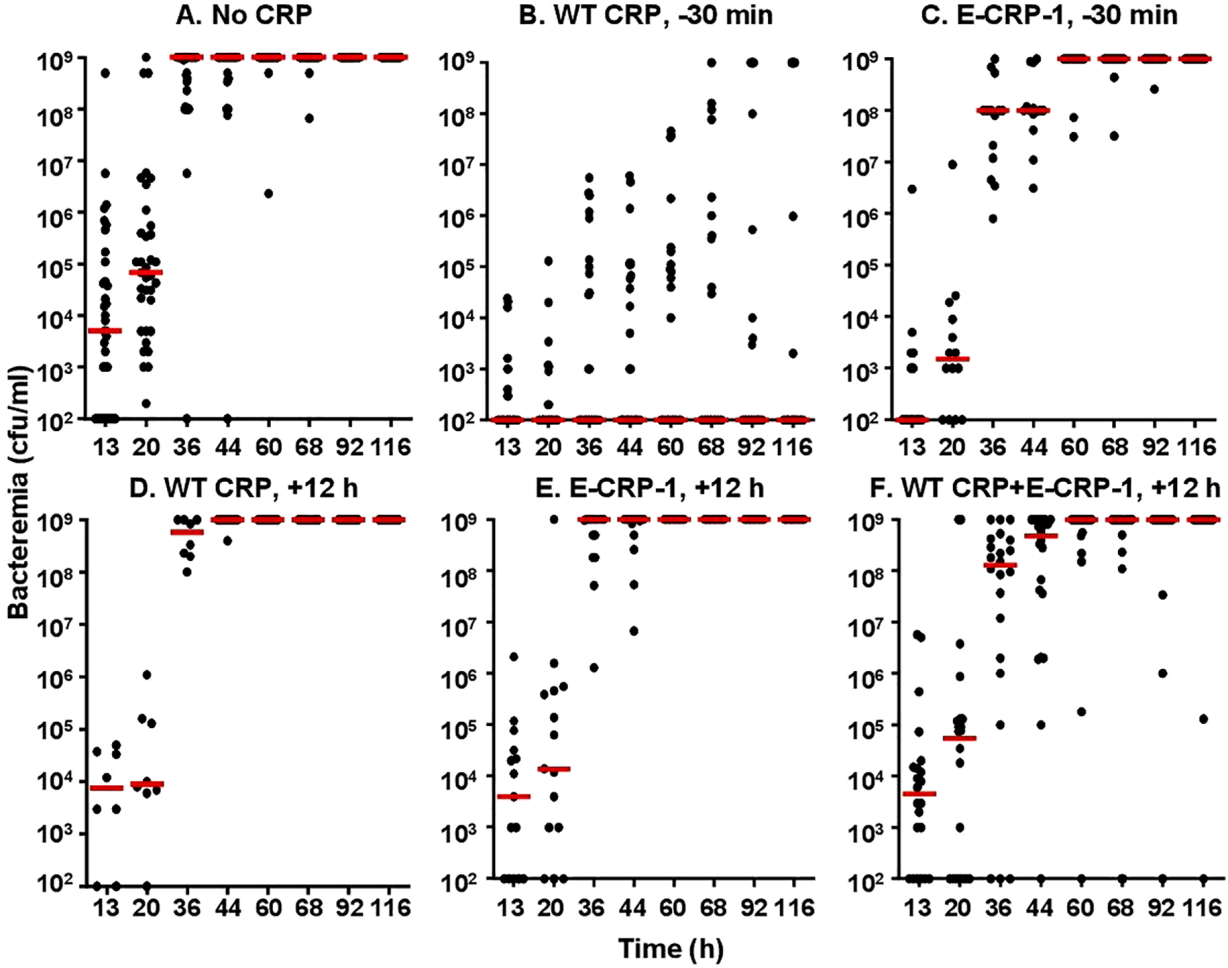
Bacteremia in CRP KO mice inoculated with pneumococci and treated with E-CRP-1 **(A–F)**. Blood was collected from each surviving mouse shown in **Figure 4**. Bacteremia was determined by plating. The bacteremia values for dead mice were recorded as 10^9^ cfu/ml. Bacteremia values of 0–100 were plotted as 100 and bacteremia values of >10^8^ cfu/ml were plotted as 10^9^ cfu/ml. The red horizontal line in each group of mice represents median bacteremia.

**Figure 6:**
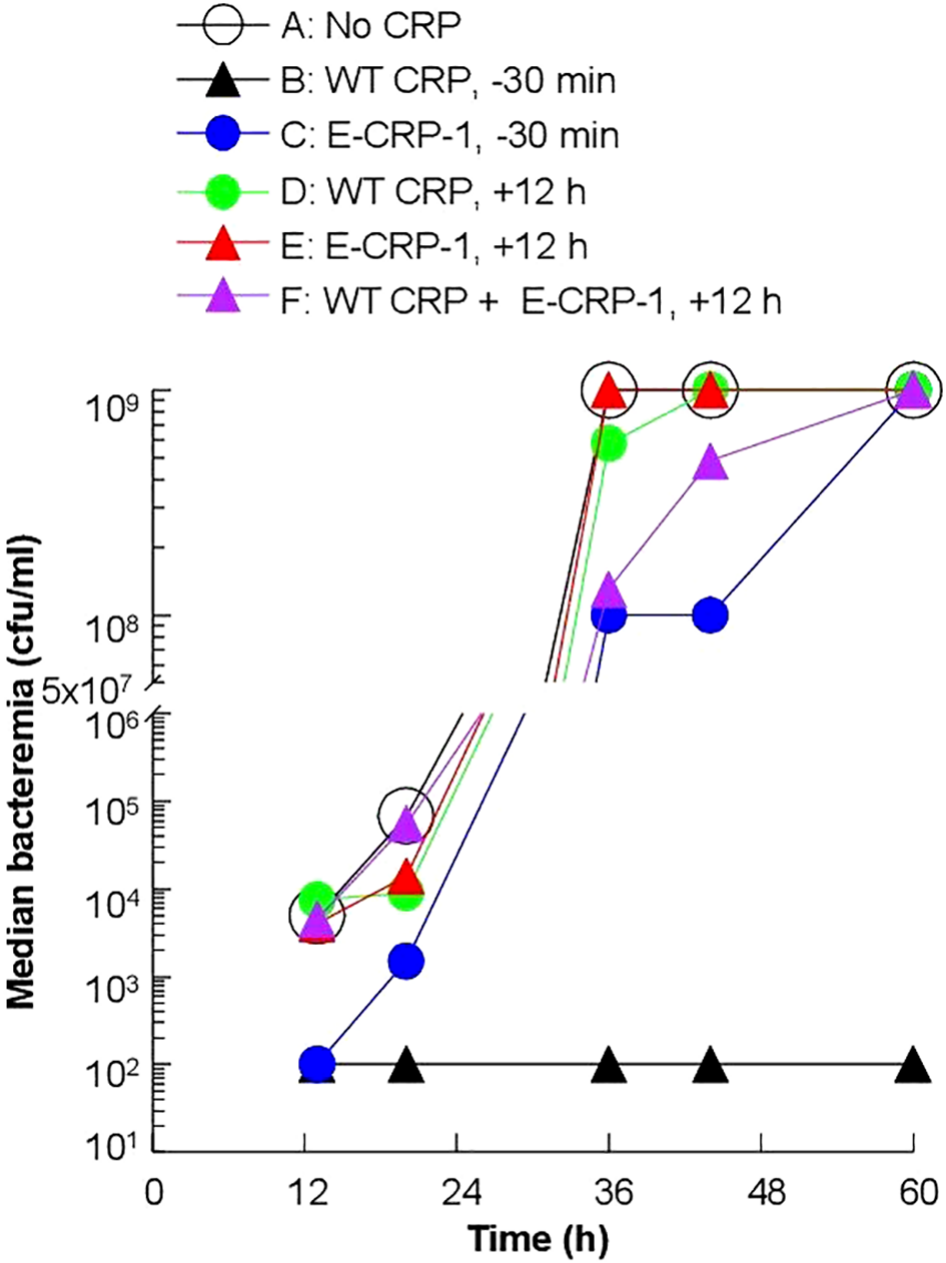
Median values of bacteremia in mice inoculated with pneumococci with and without E-CRP-1. The median bacteremia values for all six groups of mice shown in panels **(A-F)** in **Figure 5** are plotted together for comparison. For all time points, the *p*-values for the differences between groups A, C were <0.01. For all time points, the *p*-values for the differences between groups A, D and between groups A, E were >0.05. For 36 h and 44 h, the *p*-values for the difference between groups A, F were <0.01.

To determine whether the increased survival of mice was due to reduced bacteremia, we measured bacteremia in each surviving mouse in all six groups of mice (**Figure 5**). After 60 h of inoculation, bacteremia reached >10^8^ cfu/ml in all groups of mice except in mice treated with WT CRP 30 min prior to inoculation. For statistical analysis and to compare bacteremia between any two groups of mice, the median values of bacteremia (**Figure 5**) for each time point up to 60 h for all groups of mice were plotted together (**Figure 6**). The bacteremia in mice treated with either WT CRP (group D) or E-CRP-1 (group E) was not significantly different from bacteremia in untreated mice (group A). However, the bacteremia in mice treated with both WT CRP and E-CRP-1 (group F) was found to be significantly lower than bacteremia in untreated mice (group A) at time points 36 h and 44 h. Consistent with the survival curves of mice treated with E-CRP-1 alone 30 min prior to inoculation (**Figure 4**), these mice had significantly lower bacteremia than in untreated mice and at the same time, significantly higher than mice treated with WT CRP alone 30 min prior to inoculation.

### E-CRP-2 by itself protects KO mice when administered 12 h after inoculation

**Figure 7:**
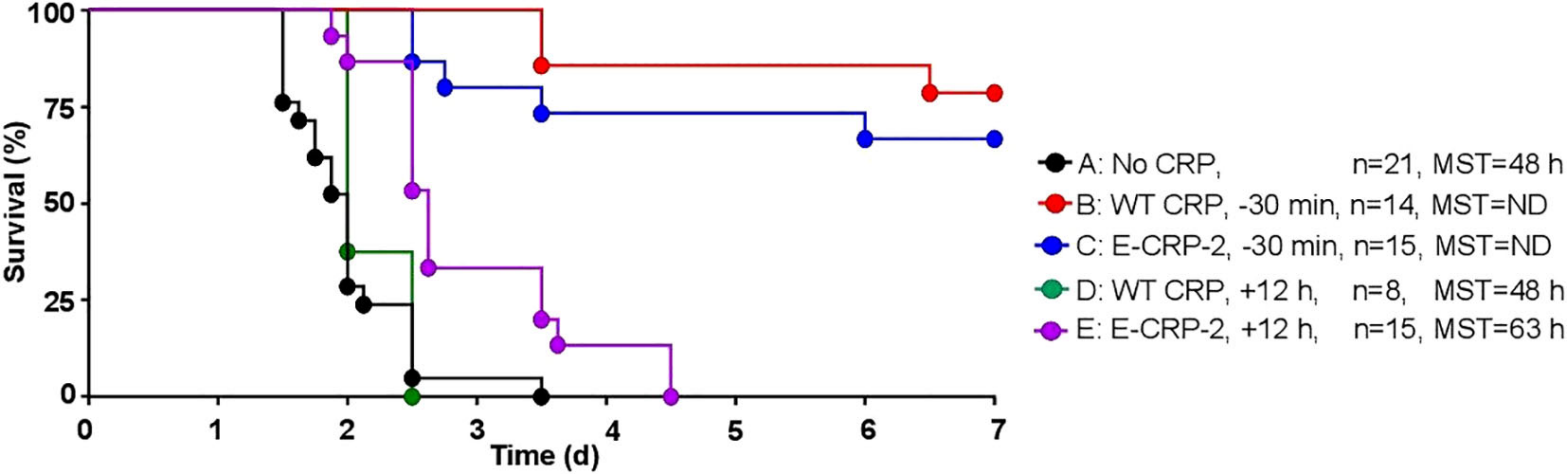
Survival of CRP KO mice inoculated with pneumococci and treated with E-CRP-2. The data are combined from two to three separate experiments with six to eight mice in each group in each experiment (n, number of mice). The MST values are shown for each group of mice (ND, MST not determined since >50% mice survived). CRP was injected 30 min prior to inoculation with pneumococci (10^7^ cfu) in groups B and C and 12 h after inoculation in groups D, E. The *p*-values for the differences in the survival curves between groups A B, A C and A E were 0.001. The *p*-value for the differences between A, D was 0.22. The *p*-value for the difference between B, C was 0.40. The *p*-values for the differences between B D and B E were <0.001. The *p*-values for the differences between C D, C E and D E were <0.005.

The protective effects of E-CRP-2 which binds to both PCh and amyloids was determined next. As shown in **Figure 7**, WT CRP was protective when administered 30 min prior to inoculation (group B) but was not protective when administered 12 h after inoculation (group D). In contrast to WT CRP, E-CRP-2 was protective irrespective of whether E-CRP-2 was administered 30 min prior to inoculation (group C) or 12 h after inoculation (group E). However, the survival curve of mice in which E-CRP-2 was administered 12 h after inoculation was found to be significantly different from both group A (no CRP) and groups B and C. When CRP was administered 12 h after inoculation, the survival times of mice treated with E-CRP-2 were found to be longer than the survival times of mice treated with WT CRP.

**Figure 8:**
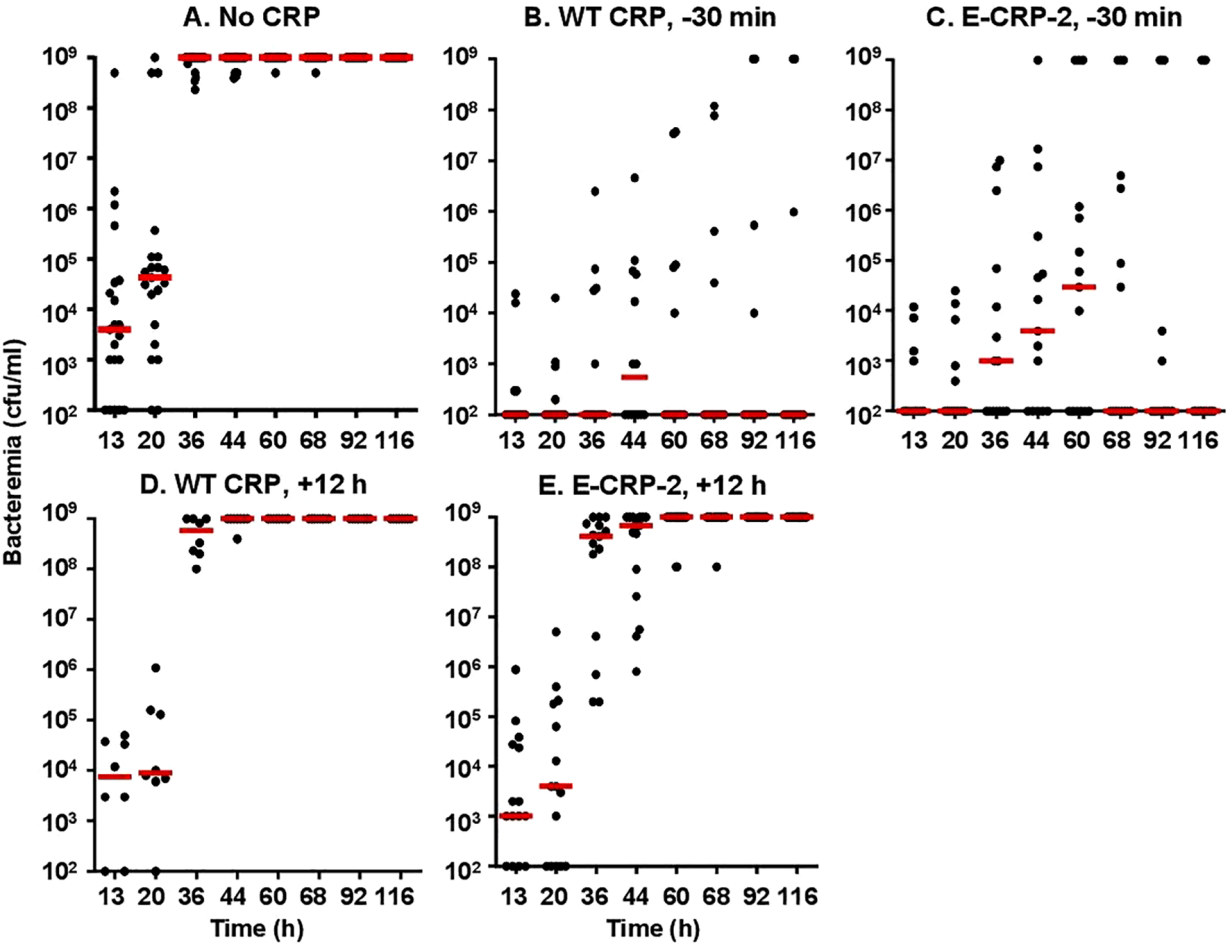
Bacteremia in CRP KO mice inoculated with pneumococci and treated with E-CRP-2 **(A–E)**. Blood was collected from each surviving mouse shown in **Figure 7**. Bacteremia was determined by plating. The bacteremia values for dead mice were recorded as 10^9^ cfu/ml. Bacteremia values of 0–100 were plotted as 100 and bacteremia values of >10^8^ cfu/ml were plotted as 10^9^ cfu/ml. The red horizontal line in each group of mice represents median bacteremia.

**Figure 9:**
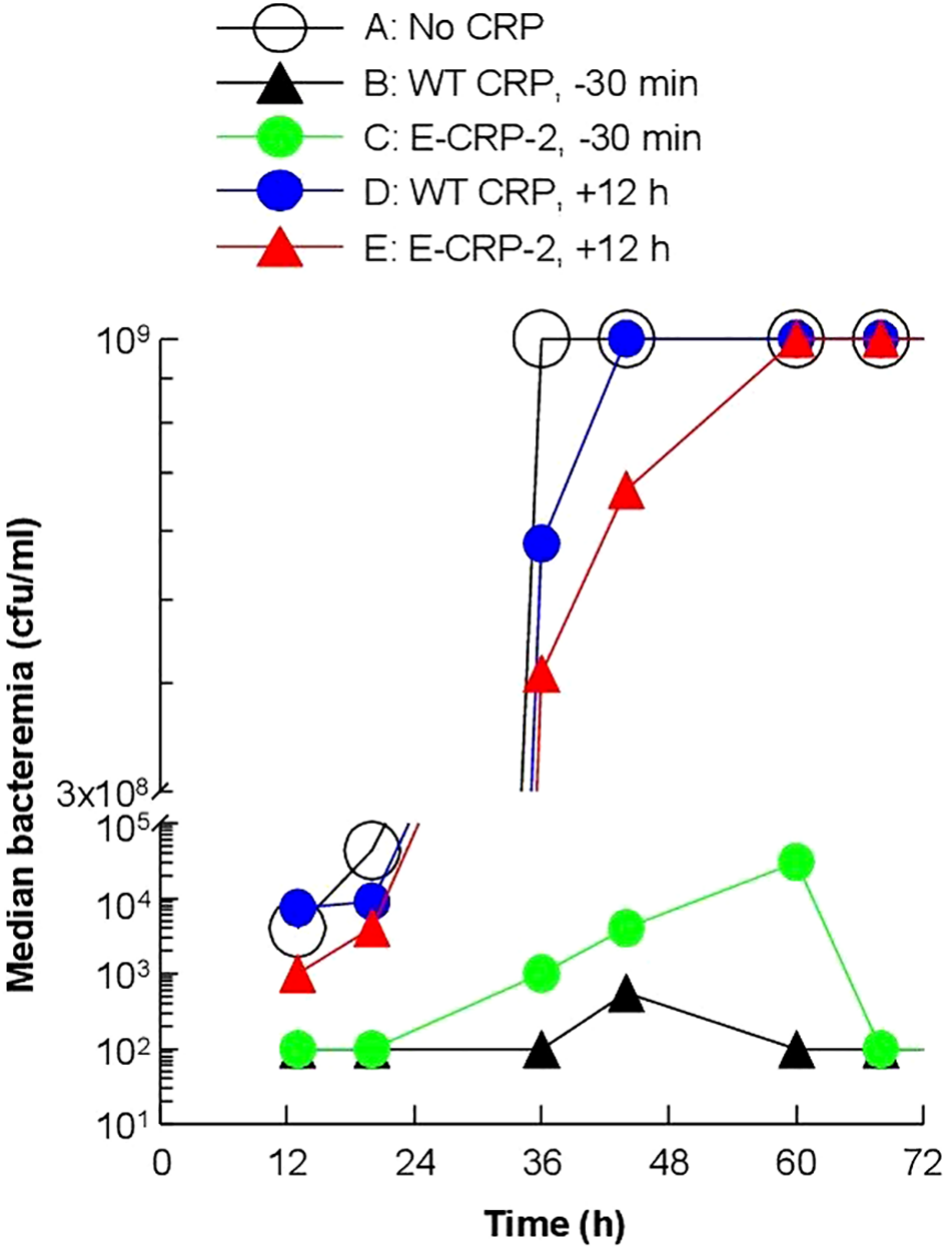
Median values of bacteremia in mice inoculated with pneumococci with and without E-CRP-2. The median bacteremia values for all five groups of mice shown in panels **(A-F)** in **Figure 7** are plotted together for comparison. For all time points, the *p*-values for the differences between groups A and C were <0.01. For all time points, the *p*-values for the differences between groups A and D were >0.05, except at 36 h where the *p*-value was <0.05. The *p* values for the difference between groups A and E for time points 13 h and 20 h were >0.05 and for 36 h and 44 h were <0.005.

Next, we measured bacteremia in each surviving mouse from all five groups of mice (**Figure 8**). After 66 h of inoculation, bacteremia reached >10^8^ cfu/ml in all groups of mice except in mice treated with either WT CRP or E-CRP-2, 30 min prior to inoculation. For statistical analysis and to compare bacteremia between any two groups of mice, the median values of bacteremia (**Figure 8**) for each time point up to 66 h for all groups of mice were plotted together (**Figure 9**). Bacteremia was significantly reduced in mice treated with either CRP species 30 min prior to inoculation for the entire duration of the experiment. Bacteremia was not reduced in mice treated with WT CRP 12 h after inoculation, except at 36 h time point. In contrast, when E-CRP-2 was administered 12 h after inoculation, bacteremia was significantly reduced sometime after 20 h post-inoculation and remained low till at least 44 h. The bacteremia data were largely consistent with the survival data shown in **Figure 7**.

### Amyloids are present on the pneumococcal surface

**Figure 10:**
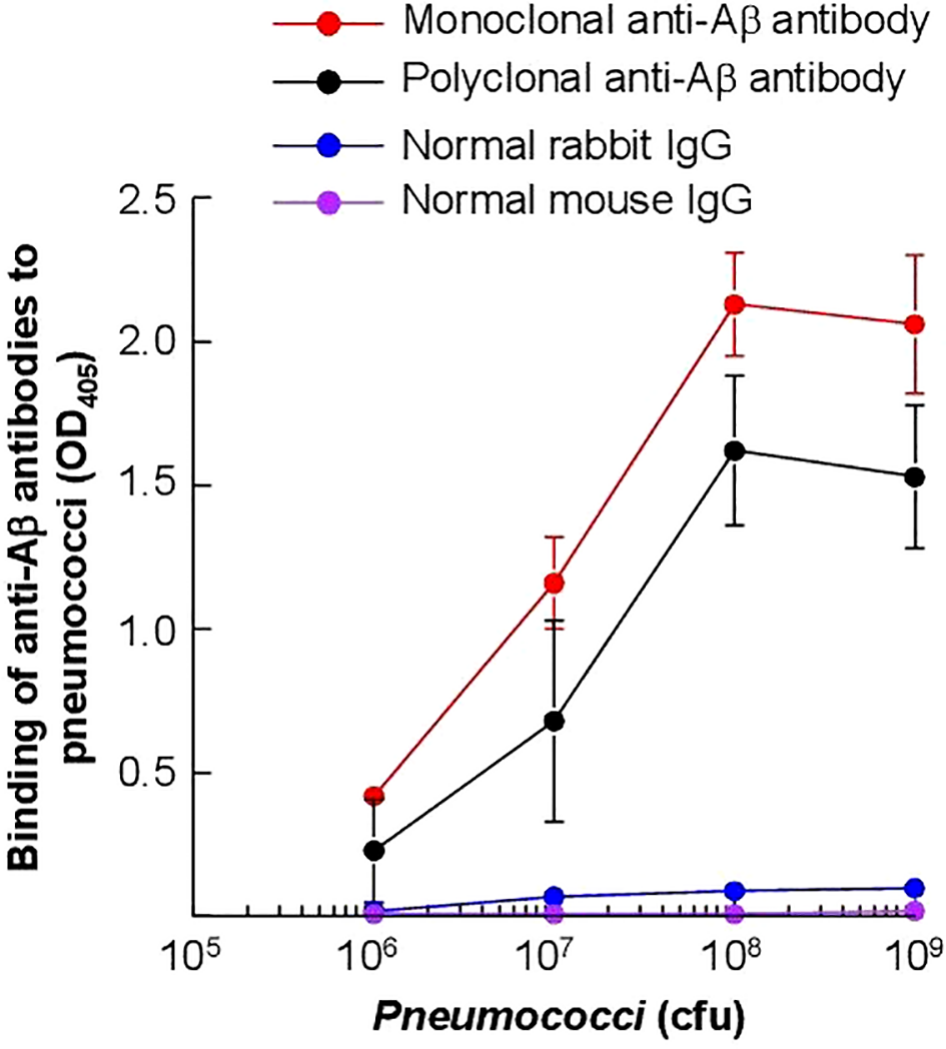
Detection of Aβ epitopes on the surface of *S. pneumoniae*. Microtiter wells were coated with pneumococci. Anti-Aβ antibodies, both monoclonal and polyclonal, were then added to the wells. Normal mouse IgG and rabbit IgG were included as controls. Bound monoclonal anti-Aβ antibodies and normal mouse IgG were detected by using HRP-conjugated goat anti-mouse IgG. Bound polyclonal anti-Aβ antibodies and normal rabbit IgG were detected by using HRP-conjugated donkey anti-rabbit IgG. The OD of the developed color was read at 405 nm. Data shown are mean ± SEM of three experiments.

First, we determined whether amyloids were present on pneumococci which were cultured *in vitro* in broth and used to inoculate mice. As shown in **Figure 10**, pneumococci were reactive with both polyclonal and monoclonal anti-Aβ antibodies. These data suggested that amyloids were already present on the surface of pneumococci even if the bacteria were not grown in the serum in the presence of complement inhibitor proteins. For this reason, we did not investigate the presence of amyloids on pneumococci which were grown *in vivo* in mice and then isolated from the blood.

### SAP levels are higher in KO mice than in WT mice

**Figure 11:**
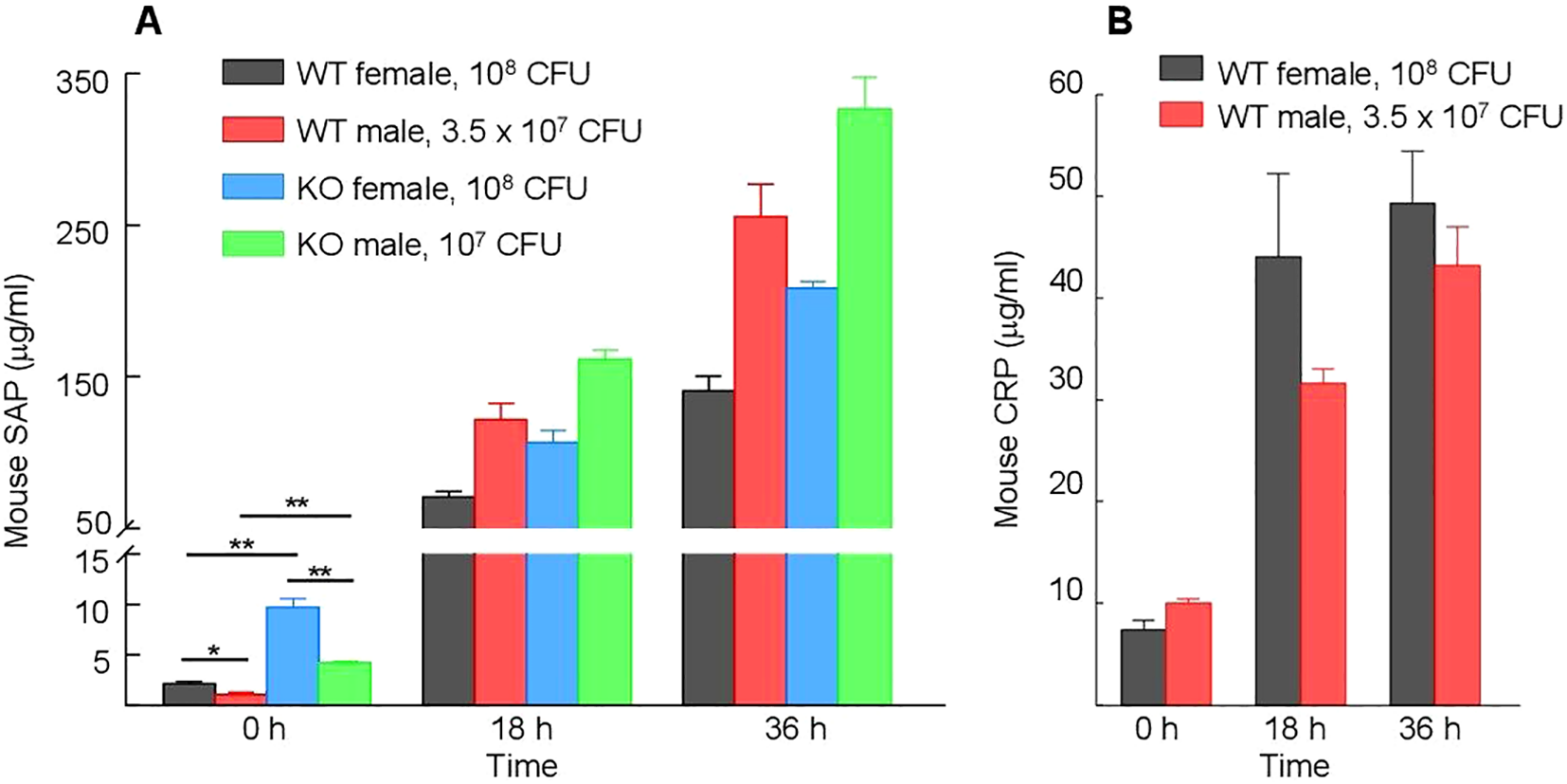
Serum levels of endogenous moCRP and SAP. Different types of mice were inoculated with different doses of pneumococci; the doses of pneumococci were chosen based on the relative susceptibility of each type of mouse to infection. Blood was collected just prior to inoculation (time zero) and at 18 h and 36 h post-inoculation. The data shown are mean ± SEM of three assays. At time zero, there were 8 mice in each group. At 18 h and 36 h time points, there were 5 mice in both groups of female mice and 8 mice in both groups of male mice, since 3 WT and 3 KO female mice died after infection. The *p-*values are shown as *p < 0.05 and **p < 0.005. **(A)** Levels of CRP in WT mice before and after inoculation. **(B)** Levels of SAP in WT and KO mice before and after inoculation.

In mice, CRP is a minor acute phase protein (50). SAP, an amyloid-binding protein, is the major acute phase protein in mice (51–54). Since the four types of mice (WT female and male, KO female and male) responded differently to the severity of infection in terms of their MST, we measured the serum levels of moCRP in WT mice and of SAP in WT and KO mice. As shown in **Figure 11A**, the basal levels of SAP in the sera (0 h) were approximately five-fold higher in KO mice than in WT mice, irrespective of the sex of mice (*p* = <0.005). The induced levels of SAP in mice after 36 h of inoculation was also higher in KO mice than in WT mice, irrespective of the sex of mice (*p* = <0.005). These data suggest that the absence of endogenous CRP can trigger acute phase response and that SAP can substitute CRP.

### Basal SAP levels are higher in female mice than in male mice

In both WT and KO mice, the basal levels of SAP were significantly higher (*p* = <0.005) in female mice than in male mice (**Figure 11A**). At 36 h post-inoculation, however, the levels of SAP were significantly higher in males than in females (*p* < 0.005), probably due to greater induction of SAP expression in male mice in response to inoculation. In WT mice, the increase in the levels of SAP was ~250-fold in male mice and ~50-fold in female mice. Similarly, in KO mice, the increase in the levels of SAP was ~80-fold in male mice and ~25-fold in female mice. In contrast to SAP, both the basal and induced serum levels of moCRP were not significantly different (*p* = >0.05) in male and female mice (**Figure 11B**).

## Discussion

WT CRP protects mice against pneumococcal infection if administered to mice prior to inoculation with bacteria and does not protect if administered a few hours after inoculation (30, 34). Thus, WT CRP is not protective against prolonged infection. We tested the hypothesis that the amyloid-binding function of structurally altered CRP is required for CRP-mediated protection against prolonged infection. In this study, we employed CRP KO mice and investigated the effects of the amyloid-binding CRP molecules E-CRP-1 and E-CRP-2 on the survival of and bacteremia in mice when administered 12 h after inoculation. There were three major findings: 1. Amyloids were detected on the surface of broth-cultured pneumococci. 2. Unlike WT CRP alone, the combination of WT CRP and E-CRP-1 was protective when administered to mice 12 h after inoculation. E-CRP-2 by itself protected mice when administered 12 h after inoculation. 3. Serum SAP levels were higher in KO mice than in WT mice. Also, the basal SAP levels were higher in female mice than in male mice and, conversely, male mice were more susceptible than female mice to infection. Thus, there was an inverse relationship between the susceptibility of mice to infection and basal SAP levels in their sera.

In a previous study, WT mice were employed to investigate the effects of E-CRP-1 on pneumococcal infection (43). It was reported that E-CRP-1 was protective in WT mice against infection when administered 12 h after inoculation. The explanation was that E-CRP-1 blocked the complement inhibitor proteins recruited by pneumococci and then endogenous moCRP could activate complement by complexing with the PCh groups on pneumococci. Subsequently, it was reported that many proteins, when immobilized on microtiter plates, expressed amyloids (21), which raised the possibility that complement inhibitors expressed amyloids once immobilized on the pneumococcal surface and that the amyloids were the ligands for E-CRP-1. In this study, we found that pneumococci cultured in broth, with no exposure to serum complement inhibitors, had amyloids on their surface. The presence of functional amyloids on bacterial surfaces has been demonstrated previously (55). The sources of the amyloids on broth-grown pneumococci are not known; however, it is possible that the surface virulence factors are amyloidogenic proteins. The possibility that E-CRP-1 directly binds to virulence factors has been raised previously based on the following findings: the binding of E-CRP-1 to pneumococci was 99% less than the binding of WT CRP to pneumococci, and that the residual binding of E-CRP-1 to pneumococci occurred in the absence of Ca^2+^ (43). Since broth-cultured pneumococci were already amyloid-positive, we did not test pneumococci isolated from infected mice for the presence of surface amyloids. However, the possibility that both the virulence factors and the complement inhibitor proteins recruited by virulence factors express amyloids still exists.

In the current study, instead of WT mice, KO mice were employed to investigate the effects of E-CRP-1 on pneumococcal infection. In this animal model, E-CRP-1 by itself was not protective but the combination of exogenous WT human CRP and E-CRP-1 was protective when administered to mice 12 h after inoculation. Like the combination of WT CRP and E-CRP-1, E-CRP-2 by itself was protective. These findings provide a proof of concept that CRP can protect against prolonged infection provided that both PCh and amyloids on the bacterial surface are occupied by either structurally altered CRP molecules mimicking E-CRP-2 or by WT CRP and structurally altered CRP molecules mimicking E-CRP-1, respectively. Although both E-CRP-1 and E-CRP-2 were protective when administered to mice 12 h after inoculation, the protection was not as good as the protection seen when CRP was administered to mice 30 min prior to inoculation. It is possible that multiple injections of WT CRP and E-CRP-1 or E-CRP-2 or higher doses of each CRP species would result in better protection than seen with the single injection of structurally altered CRP reported here.

It is not known whether structurally altered CRP mimicking the ligand-binding properties of E-CRP-1 or E-CRP-2 is formed at sites of inflammation *in vivo* during pneumococcal infection. Since CRP mutants E-CRP-1 and E-CRP-2 mimic the ligand-binding properties of acidic pH-treated CRP and H_2_O_2_-treated CRP, these mutants provide us with a tool to identify the actual CRP species present at sites of inflammation. E-CRP-1 and E-CRP-2 can be used to generate a library of monoclonal antibodies which can then be screened to identify the antibodies which neither react with WT CRP nor with monomeric CRP. The antibodies specific for E-CRP-1 and E-CRP-2 can then be employed to detect structurally altered pentameric CRP *in vivo* and to locate the sites where WT CRP is converted to amyloid-binding forms of CRP.

Our data suggest that the recognition of bacterial amyloids by structurally altered CRP is critical for protection against prolonged infection. In theory then, any amyloid-binding protein administered to mice should give the results similar to that of E-CRP-1 and E-CRP-2. Since SAP is an amyloid-binding protein, SAP should be able to bind to bacterial amyloids and contribute to CRP-mediated protection. It is also possible that the binding of SAP to bacterial amyloids reduces the virulence of pneumococci. It has been shown previously that SAP binds to pneumococci and activates complement (56); however, it has been suggested that the ligands of SAP on pneumococci were surface carbohydrates since SAP also binds to carbohydrates.

While developing the KO mouse model for this study, we noted sex-specificity in the expression of the SAP gene, in the susceptibility of both WT and KO mice to infection and in the SAP levels in the sera from both WT and KO mice. There was no sex-specificity in the expression of the moCRP gene, although it has been reported previously that the expression of the human CRP transgene in mice was sex-specific (28, 57). We found that female mice were less susceptible than male mice to infection; these results are consistent with a previously published report that, in both animals and humans, males are generally more susceptible than females to bacterial infections and that women have stronger immune responses to foreign antigens than men (58). We propose that female mice were less susceptible than male mice to infection because female mice had higher basal SAP levels in their sera than in the sera of male mice. The inverse relationship between the susceptibility of mice to infection and the basal levels of SAP in the sera further suggests that SAP plays a role in protecting against pneumococcal infection.

As described above, the capability of SAP to bind amyloids and the relationship between basal SAP levels and susceptibility to infection both suggested the involvement of SAP in protection against pneumococcal infection. Another evidence to support the role of SAP in pneumococcal infection came from the data on SAP levels seen in KO mice which was higher than in WT mice. The higher expression of the SAP gene in the absence of the CRP gene supports our interpretation that SAP plays a role in protecting against pneumococcal infection. Indeed, employing SAP KO mice, it has been shown previously that mouse SAP participates in protection against pneumococcal infection (56).

We conclude that the protection against prolonged pneumococcal infection involves conformational changes in CRP, binding of CRP to both PCh and amyloids on the pneumococcal surface, and complement activation by PCh-complexed CRP. The two recognition functions of CRP, PCh-binding and amyloid-binding, can be exhibited by two different CRP molecules as exemplified here by the combination of WT CRP and E-CRP-1 or by a single structurally altered CRP molecule if the PCh-binding function is retained in the structurally altered form as exemplified here by E-CRP-2. The combined data reported here and published previously (56) suggest that SAP cooperates with CRP in protection against pneumococcal infection. We propose that, in circulation, CRP cooperates with SAP to reduce bacteremia. At sites of inflammation in the organs, where the conformation of CRP can be altered, WT CRP cooperates with structurally altered CRP to reduce bacterial load in the organs. Future investigations employing double SAP KO and CRP KO mice would provide definitive proof for the cooperation between SAP and CRP in controlling pneumococcal infection.

## Data Availability

All data generated for this study is presented in this article. Further inquiries can be directed to corresponding authors.
